# Mental well-being and work capacity: a cross-sectional study in a sample of the Swedish working population

**DOI:** 10.1186/s12889-025-24015-1

**Published:** 2025-09-09

**Authors:** Agneta Blomberg, Gunnel Hensing, Monica Bertilsson, Carin Staland-Nyman, Christian Ståhl, Lisa Björk

**Affiliations:** 1 School of Public Health and Community Medicine, Institute of Medicine, Sahlgrenska Academy, University of Gothenburg, PO Box 453, Gothenburg, SE-405 30 Sweden; 2 School of Health and Welfare, Halmstad University, Halmstad, Sweden; 3 Department of Behavioural Sciences and Learning, Division of Education and Sociology, Linköping University, Linköping, Sweden; 4 Department of Sociology and Work Science, University of Gothenburg, Gothenburg, Sweden; 5 Institute of Stress Medicine, Region Västra Götaland, Gothenburg, Sweden

**Keywords:** Mental well-being, Work capacity, Work participation, Common mental disorders, Mental health, Working population

## Abstract

**Background:**

Mental health problems are common in the working-age population. More knowledge is needed on how to support work participation and reduce sickness absence. The objective of the study was to estimate the distribution of mental well-being and work capacity in women and men in a working population and assess the association between mental well-being and work capacity, while adjusting for sociodemographic characteristics, health status, and working positions.

**Methods:**

Cross-sectional data were collected through an online survey distributed to individuals who were currently working. The study population consisted of 8462 employees (58% women). The WHO-5 Mental Well-being Index (scale ranging from 0 to 100 with higher scores representing a better mental well-being) and the Capacity to Work Instrument (C2WI) (scale ranging from 14 to 56 with higher scores representing a more strained work capacity) were used. Univariable and multivariable linear regressions were used to assess the associations between self-perceived mental well-being and capacity to work, adjusting for sociodemographic characteristics, health status, and working positions.

**Results:**

Low self-perceived mental well-being and strained work capacity were more common among women, particularly younger aged (18–34 years). Poor health status was associated with strained work capacity in both men and women. Regression analyses showed that lower self-perceived mental well-being was significantly associated with strained work capacity. Among women, the fully adjusted model showed a regression coefficient (B) of − 0.253 (95% CI: −0.264 to − 0.242); among men, it was − 0.225 (95% CI: −0.237 to − 0.213).

**Conclusions:**

This study, focusing on a currently working population, identified disparities in self-perceived mental well-being and work capacity across gender and age groups. These findings underscore the importance of early workplace interventions to support mental well-being and work capacity in these sub-groups. Notably, the association between the WHO-5 and C2WI may be partly attributable to item-level overlap, as certain C2WI items may capture symptoms related to mental health. This potential overlap should be considered when interpreting the findings.

**Supplementary Information:**

The online version contains supplementary material available at 10.1186/s12889-025-24015-1.

## Background

Enabling a larger share of the working-age population to participate in paid employment has benefits for both individuals and society as a whole [1, 2]. From the perspective of the individual, the core rationale for work participation (WP) is to provide for oneself and, in many cases, for one’s family and relatives. From a societal perspective, there is a need to secure the production of goods and services, as well as the provision of welfare services [3]. Despite the relevance of better understanding WP, few studies have focused on the factors that facilitate WP with mental health problems. Instead, most research has examined risk factors for non-WP, such as sickness absence and disability retirement [4–6].

The definition by Lagerveld et al. (2010) [7] states that WP is the capability and opportunity to engage in productive work within the workforce while fulfilling one’s work role. Mental health is an important capability, as it supports WP. In contrast, common mental disorders (CMD), including less well-defined symptoms, negatively impact WP [8]. Although CMD and related symptoms are prevalent among working-age individuals [9, 10], most of those affected continue to work [11]. This may be the result of cognitive behavioural therapy, which has proven effective in reducing symptoms [12]. In fact, interventions to promote return to work in sick-listed individuals with psychiatric disorders have been efficient in relieving symptoms, while return to work has been less successful [13]. Thus, WP does not necessarily follow symptom recovery. Another significant function for WP is work capacity, serving both as a determinant and a resource in shaping work-life trajectories [14]. However, the capacity to work phenomena is complex, encompassing several dimensions, including individual abilities and resources [15, 16], as well as the organizational demands and resources required for work performance [17, 18]. Moreover, work capacity is contingent upon age, education, health, competence, values, and attitudes [19–21], as well as the specific demands of the work tasks and the characteristics of the work environment [18, 22].

Furthermore, the complexity of measuring work capacity presents significant challenges. The most utilized metric in studies of the working population is the generic Work Ability Index (WAI) [19]. More specific measures have been developed to address mental disorders within clinical populations, i.e., persons who seek care for mental health problems [23, 24]. The Capacity to Work Index (C2WI) was developed as a CMD-specific measure, focusing not only on the fact *that* work capacity may be affected, but also *why* work capacity may be affected [25]. The objective of this study was to estimate the distribution of mental well-being and work capacity in women and men in a working population and assess the association between mental well-being and work capacity, while adjusting for sociodemographic characteristics, health status, and working positions.

## Methods

An epidemiological cross-sectional study design was used, and data were collected from autumn 2021 until summer 2022 through an online questionnaire (Additional file 1).

### Recruitment process

The recruitment process for the data collection started in spring 2021. Contact was made with Swedish companies (*n* = 15) in the private sector and with various trade unions (*n* = 15), primarily representing employees working in the private sector, to reach currently working individuals. Information about the study was provided at follow-up meetings and through emails. Private employers who agreed to support the project provided the employees’ email addresses. Unionized employees were invited to the survey through their trade union representatives via newsletters and emails. Municipalities and regional authorities (*n* = 136) were contacted to recruit participants working in the public sector in the same manner. The number of individuals recruited was insufficient to address the research questions. Therefore, a web panel (the Norstatpanel) was utilized to expand the sample size. The Norstatpanel consists of individuals who have been randomly contacted via telephone and subsequently agreed to participate in the overall panel. The panel is broadly representative of the population and has a nationwide scope [26]. Participants were recruited based on their match to the specified target population, defined as individuals aged 18 years or older who were currently working.

### Data collection

The data were collected from three different sources at three time points. The initial data collection commenced in November 2021, and a second data collection phase was initiated in April 2022. The third data collection based on the Norstatpanel commenced in May 2022. Detailed information about the study, including ethical considerations and unique links to the questionnaire, was sent directly to employees in private companies and public organizations via email. Open-ended links were sent to union members via trade unions through newsletters and emails. The survey was concluded in August 2022. Two reminders were sent during each data collection period. Respondents were required to identify themselves using an e-ID to participate in the survey. The number of respondents from the first data collection was 397; there were 3764 and 6119 respondents from the second and third data collections, respectively, resulting in a total of 10 280 respondents.

### Study population

**Fig. 1:**
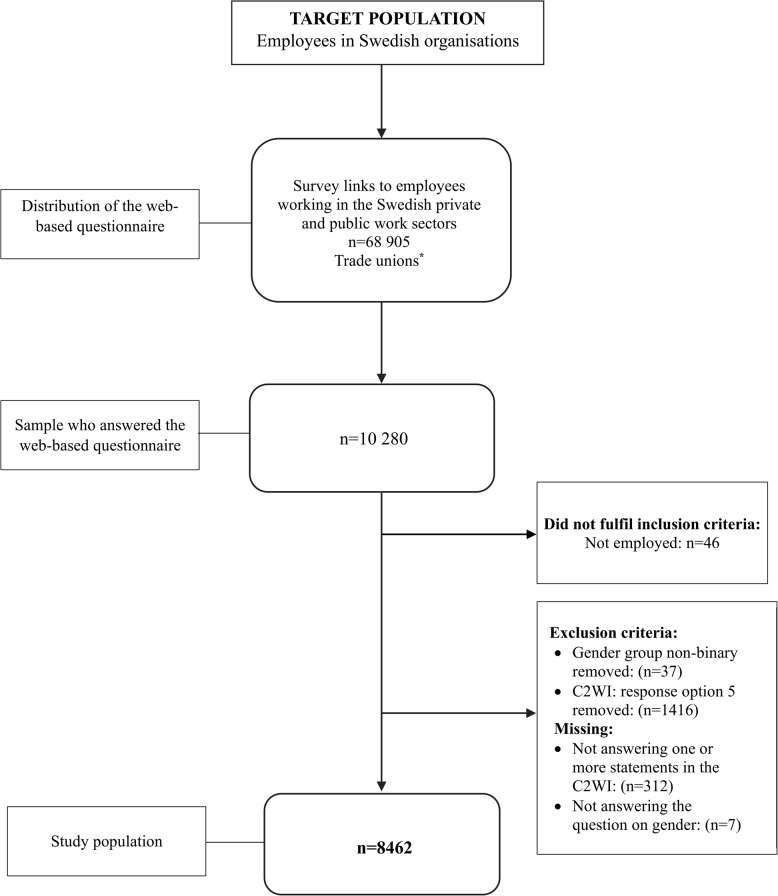
Flowchart of the inclusion procedure and response rates based on the Swedish “Work Participation and Mental Health at Work” (ADAPT) project, 2021-2022 *The trade unions distributed survey links to trade union members through newsletters and e-mail

The target population consisted of individuals aged 18–75 who reported being currently working. The inclusion procedure is presented in Fig. 1. Respondents not currently in active labour were excluded (*n* = 46). The data used in the descriptive statistics and statistical analyses were based on excluding cases with missing data for gender (*n* = 7) and the non-binary group (*n* = 37). For the Capacity to Work instrument (C2WI), we excluded response option 5 in any of the statements, as the combination “don’t know/not relevant” made it unclear (*n* = 1416). Those with items missing at random (*n* = 191) and those with internal data missing (*n* = 121) were also excluded. The proportion of women (58%) and men (42%) in the final study sample (*n* = 8462) was compared with that of the general working population and found to be similar (54% women and 46% men) [27].

### Survey and instruments

The online survey included questions regarding sociodemographic characteristics and utilized instruments relevant for assessing the association between self-perceived mental well-being and work capacity (Additional file 1).

### Dependent variable

The C2WI was developed to capture the different aspects of CMD-related work capacity [25]. In this study, it was applied as an indicator of strained work capacity. Capacity to work was measured through 14 statements (Additional file 2), such as “Thinking has been tough and slow”, “I have had to choose to not do free-time activities to have energy to work”, and “I have felt wound up”. Each statement had four response options: “not at all” (1), “to a low degree” (2), “to a moderate degree” (3), “to a high degree” (4). The minimum score for the C2WI was 14 (score 1 for 14 items) and the maximum score was 56 (score 4 for 14 items). For the descriptive statistics, scores were dichotomized at the 75th percentile (≥ 32) (range 14–56). The higher the scores, the more strained the work capacity. A continuous variable was used for the regression analyses. Cronbach’s alpha was 0.92.

### Independent variable

The World Health Organization’s 5-item Well-Being Index (WHO-5) is a generic tool designed to measure subjective mental well-being [28], and the Swedish version of the WHO-5 has been deemed psychometrically sound [29]. The WHO-5 uses positively framed statements: ”I have felt cheerful and in good spirits”, “I have felt calm and relaxed”, “I have felt active and vigorous”, “I woke up feeling fresh and rested”, and “My daily life has been filled with things that interest me”, with response options ranging from “at no time” (0) to “all of the time” (5) on a 6-point Likert scale. Scores were summed and multiplied by four to form a scale from 0 (lowest) to 100 (highest). For the descriptive statistics, scores were dichotomized at the 25th percentile (≤ 44). Lower scores indicate reduced mental well-being. A continuous variable was used in the regression analysis. Cronbach’s alpha was 0.89.

### Covariates

Sociodemographic characteristics such as age, education, and occupational class influence working conditions and access to health-supporting resources, which are associated with risks of work disability [14–16]. Mental well-being, general health status, and the presence of long-term health conditions are closely linked to perceived work capacity [18–20]. Finally, working positions such as work sector, managerial position and working time were added [21, 22].

The respondent’s birth year was recalculated into age, ranging from 18 to 74 years, and recoded into the categories: 18–34, 35–54, and 55–74 years. In Sweden, the official retirement age is 67 years. Thereafter, an employee may continue working until 69 years of age, and beyond that age, upon mutual agreement between the employee and the employer. Level of education was recoded into four categories: compulsory or lower secondary school, upper secondary school, post-secondary school, and university or higher education. Occupational class was determined using the Swedish Standard for Swedish Occupational Classification (SSYK2012) [30]. SSYK is based on a four-level hierarchy and was recoded into four categories: non-manual high-skilled; non-manual low-skilled; manual high-skilled, and manual low-skilled as an indicator of occupational class.

General health status was assessed with one question from the from the 36-Item Short Form Survey (SF-36) [31]: “In general, how would you describe your health?” with response options ranging from “very good” (1) “ good” (2), “fair” (3), “poor” (4) and “very poor” (5). The categories were recoded into three levels: low (“poor, very poor” (1), moderate (“fair”) (2), and high (“good, very good”) (3).

A question on long-term health conditions encompassed 12 different disease groups. Respondents could select multiple disease groups. We created four groups: (i) none of the listed disease groups, (ii) mental health conditions only, (iii) mental health conditions combined with other long-term health conditions, and (iv) other long-term health conditions only [32].

Work sector was categorized as public, private, or other. The category “other” was deemed too small (*n* = 117) and was therefore excluded.

A managerial position provides occupational prestige and increased job autonomy, both of which positively affect job satisfaction [33]. At the same time, gender inequality regarding the benefits associated with managerial roles has been identified and was therefore included. Managerial position was measured as a binary variable (“yes” or “no”). Female employees are more likely to work part-time than men [34]. This phenomenon is more prevalent in female-dominated occupations and is partly driven by occupational segregation [35]. Working part-time or full-time was measured as a binary variable (“yes” or “no”).

### Statistical analysis

Data analyses were performed using IBM SPSS Statistics 29.0.2.0 [36]. The baseline characteristics are presented as proportions with 95% confidence intervals (95% CI).

Descriptive and inferential methods were employed to assess mental well-being and work capacity. Building on previous research on mental well-being in a population sample, this study adopted the same approach, using a cutoff score at the first quartile [37, 38]. As the C2WI is a newly developed instrument, we applied the same method. With a high score reflecting a negative outcome, the scale was dichotomized at the third quartile (Additional files 3 and 4).

Univariable linear regression was conducted to examine the individual associations between mental well-being, each covariate, and work capacity. This approach allows for an initial assessment of the strength and direction of the relationship before adjusting for potential confounders in the multivariable models. The assumption of linearity between the continuous independent variable and the dependent variable was assessed using residual plots and found to be acceptable. The regression analyses were performed with the C2WI as the dependent variable and mental well-being (WHO–5) as the independent variable. Both entered as continuous variables. Age, level of education, general health, long-term health conditions, occupational class, work sector, managerial position and working time were entered as categorical variables. Long-term health conditions were grouped into two groups: No (none of the listed disease groups) and Yes (mental health conditions only and mental health conditions combined with other conditions). Long-term health conditions only were excluded.

In Model I, adjustments were made for sociodemographic characteristics. In Model II, adjustment was made for general health, long-term health conditions, and the variables in Model I. Based on regression analysis, we present the unstandardized regression coefficient (B), and the corresponding 95% CIs.

When tested with Chi2 analysis, we found a statistically significant difference in the distribution of work capacity scores between genders. In addition, it is well-documented that industrial countries reflect a traditional divide through occupational segregation, which warrants analytical consideration [35, 39]. The normality of the residuals was confirmed by examining the variation inflation factors and tolerances. No problems with multicollinearity were noted between the variables.

## Results

The distribution of the background characteristics is presented in Table 1. A total of 8462 individuals participated in the study of which 4905 were women (58%) and 3557 were men (42%). The majority of participants, 66% of women and 65% of men, worked in non-manual, high-skilled jobs. Women worked predominantly in the public sector (68%), while men worked in the private sector (62%).

**Table 1:** Study population stratified by gender (*n* = 8462), 2021–2022, the Swedish “work participation and mental health at work” research project

Characteristics	Women (*n* = 4905) %	Men (*n* = 3557) %
*Age groups*		
18–34 years	24	22
35–54 years	51	50
55–74 years	25	28
Missing (4)		
*Level of education*		
University or higher (≥ 16 years)	54	40
Post secondary school (13–15 years)	10	11
Upper secondary school (10–12 years)	27	35
Compulsory/lower secondary school (≤ 9 years)	9	14
Missing (3)		
*Occupational classification*		
Non-manual, high-skilled	66	65
Non-manual, low-skilled	6	2
Manual, high-skilled	5	19
Manual, low-skilled	23	14
Missing (87)		
*Managerial position*		
Yes	19	28
No	81	72
Missing (2)		
*Worktime*		
Yes, full-time	78	92
No, part-time	22	8
Missing (7)		
*WHO-5 Mental Well-being Index* (range, 0–100)		
Mental well-being above Q1: ≥45	69	79
Mental well-being below Q1: ≤44	31	21
Missing (48)		
*SF-36 General Health*		
Good/very good general health	64	72
Moderate general health	28	22
Poor/very poor general health	8	6
Missing (38)		
*Long-term health conditions*		
No	36	47
Yes, mental health conditions, only	5	4
Yes, mental health conditions and other	15	6
Other long-term health conditions	44	43
Missing (89)		
*Work sector*		
Public sector	68	38
Private sector	32	62
Missing (3)		

A larger proportion of women than men reported mental well-being scores ≤ 44 (Table 1) and this gender difference was also found between age groups, educational levels and occupational classes (Additional file 3). Work capacity scores ≥ 33 were reported by 30% of women and by 18% of men. Consistent differences between women and men were found in age groups, educational levels and occupational classes (Additional file 4). The most pronounced differences in proportion reporting scores ≥ 33 were found between women and men in the youngest age groups (18–34 years) and between women and men with university education.

A negative association was observed between mental well-being (WHO-5) and capacity to work (C2WI) among women in the two models (Table 2). In Model 1 [adjusted for age, educational level, occupational class, managerial position and working time], higher scores on C2WI were significantly associated with lower values on WHO-5 (B = − 0.317; 95% CI: − 0.326 to − 0.307). After adjusting for general health and long-term mental health conditions in Model 2, the strength of the association was attenuated but remained significant (B = − 0.253; 95% CI: − 0.264 to − 0.242). These findings suggest that while other factors partly account for the relationship, a robust inverse association between mental well-being and capacity to work persists in this sample of women even after adjusting for potential confounders.

**Table 2:** Multivariable linear regression analysis investigating the association between mental well-being* and capacity to work* in currently working women (*n* = 4905) in the Swedish research project “work participation and mental health at work”, 2021–2022

	Model I^a^		Model II^b^	
**Variables**	**B**	**95% CI**	**B**	**95% CI**
*WHO-5 Mental Well-being Index*, (range, 0–100)*	–0.317	(–0.326;–0.307)	–0.253	(–0.264;–0.242)
*Age groups*				
18–34 years (Ref.)				
35–54 years	–1.345	(–1.824;–0.865)	–1.246	(–1.711;–0.781)
55–74 years	–1.894	(–2.458;–1.329)	–1.728	(–2.280;–1.177)
*Level of education*				
University or higher (≥ 16 years) (Ref.)				
Post secondary (13–15 years)	–0.332	(–1.022;0.357)	–0.664	(–1.331;0.003)
Upper secondary (10–12 years)	–0.585	(–1.133;–0.037)	–0.812	(–1.340;–0.283)
Lower secondary or less (≤ 9 years)	–1.487	(–2.287;–0.686)	–1.614	(–2.387;–0.842)
*Occupational classification*				
Non-manual, high-skilled (Ref.)				
Non-manual, low-skilled	–0.594	(–1.483;0.296)	–0.512	(–1.370;0.346)
Manual, high-skilled	–0.693	(–1.604;0.218)	–0.614	(–1.492;0.263)
Manual, low-skilled	0.725	(0.136;1.314)	0.436	(–0.133;1.005)
*Managerial position*				
Yes (Ref.)				
No	–0.238	(–0.739;0.262)	–0.401	(–0.885;0.083)
*Working time*				
Yes, full-time (Ref.)				
No, part-time	0.737	(0.259;1.214)	0.280	(–0.184;0.745)
*SF-36 General Health*				
Good/very good general health (Ref.)				
Moderate general health			2.633	(2.147;3.118)
Poor/very poor general health			5.701	(4.867;6.536)
Long-term health conditions**				
No (Ref.)				
Yes, mental health conditions			2.515	(1.993;3.037)

*Abbreviations*: *B* Unstandardized Coefficient, *CI* Confidence Interval, *Ref.* Reference category

*Higher scores on the Mental Well-being scale indicate better mental well-being while higher scores on the C2WI indicate a more strained work capacity

**Long-term health conditions were divided into “No”– none of the disease groups and “Yes” - mental health conditions only and mental health conditions combined with other long-term health conditions. Other long-term health conditions were not included in this analysis

^a^Model I: adjusted for age, level of education, occupational classification, managerial position, and working time

^b^Model II: adjusted for model I, general health status, and long-term mental health conditions

A negative association between WHO-5 and C2WI was found also in men (Table 3). In Model 1, adjusted for age, educational level, occupational class, managerial position and working time, higher scores on C2WI were significantly associated with lower values on WHO-5 (B = −0.277; 95% CI:–0.288 to–0.267). In model 2 we adjusted for general health and long-term mental health conditions. The strength of the association was attenuated but remained significant (B =–0.225; 95% CI:–0.237 to–0.213). Thus, also in men our findings suggest an inverse association between mental well-being and capacity to work even after adjusting for potential confounders.

**Table 3:** Multivariable linear regression analysis investigating the association between mental well-being* and capacity to work* in currently working men (*n* = 3557) in the Swedish research project “work participation and mental health at work”, 2021–2022

	Model I^a^		Model II^b^	
**Variables**	**B**	**95% CI**	**B**	**95% CI**
*WHO-5 Mental Well-being Index*, (range, 0–100)*	–0.277	(–0.288;–0.267)	–0.225	(–0.237;–0.213)
*Age groups*				
18–34 years (Ref.)				
35–54 years	–1.701	(–2.224;–1.157)	–1.864	(–2.390;–1.338)
55–74 years	–2.032	(–2.657;–1.407)	–2.256	(–2.864;–1.648)
*Level of education*				
University or higher (≥ 16 years) Ref.)				
Post secondary (13–15 years)	0.545	(–0.192;1.282)	0.338	(–0.375;1.050)
Upper secondary (10–12 years)	–0.617	(–1.151;–0.083)	–0.707	(–1.223;–0.191)
Lower secondary or less (≤ 9 years)	–0.527	(–1.277;0.223)	–0.774	(–1.500;–0.048)
*Occupational classification*				
Non-manual, high-skilled (Ref.)				
Non-manual, low-skilled	–0.884	(–2.413;0.644)	–1.078	(–2.556;0.401)
Manual, high-skilled	0.135	(–0.490;0.761)	0.200	(–0.404;0.805)
Manual, low-skilled	1.333	(0.650;2.017)	0.915	(0.252;1.579)
*Managerial position*				
Yes (ref.)				
No	–1.016	(–1.503;–0.528)	–1.222	(–1.694;–0.749)
*Working time*				
Yes, full-time (Ref.)				
No, part-time	0.588	(–0.214;1.390)	0.069	(–0.711;0.848)
*SF-36 General Health*				
Good/very good general health (Ref.)				
Moderate general health			2.125	(1.562;2,688)
Poor/very poor general health			4.180	(3.160;5.199)
Long-term health conditions**				
No (Ref.)				
Yes, mental health conditions			4.169	(3.401;4.936)

*Abbreviations*: *B* Unstandardized Coefficient, *CI* Confidence Interval, *Ref.* Reference category

*Higher scores on the Mental Well-being scale indicate better mental well-being while higher scores on the C2WI indicate a more strained work capacity

** Long-term health conditions were divided into “No”– none of the disease groups and “Yes” - mental health conditions only and mental health conditions combined with other long-term health conditions. Other long-term health conditions were not included in this analysis

^a^Model I: adjusted for age, level of education, occupational classification, managerial position, and working time

^b^Model II: adjusted for model I, general health status, and long-term mental health conditions

## Discussion

This study was based on currently working individuals to gain more knowledge on work participation in individuals with lower mental well-being [11]. We found persistent gender-based disparities in the distribution of mental well-being and work capacity. We also found that lower mental well-being was associated with a more strained capacity to work in both women and men.

That lower mental well-being was found to be more prevalent among women than among men, is in line with previous findings [40, 41]. Added to the burden of lower mental well-being, we found that a higher proportion woman than men reported strained capacity to work. It is not possible from this cross-sectional study to draw any conclusions on how mental well-being and capacity to work is developed but a dynamic interplay can be assumed [25]. In future studies it would be interesting to follow if mental well-being, capacity to work or the two combined best predict future work participation. It was beyond the aim of this study, but it is well known that perceived stress at work is associated with CMD and sickness absence [7]. Our findings call for initiatives to develop organisational stress reduction at workplaces since lower mental well-being and strained capacity to work was found in both women and men. Given the higher proportion in women, female-dominated workplaces could gain most from such efforts.

Another finding was the age gradient in mental well-being and work capacity, which was specifically evident in capacity to work among women. In fact, it was a bit unexpected that women in the youngest age group had the highest proportion (37%) reported strained work capacity (C2WI scores ≥ 33). To date, much of the research on work capacity has focused on the aging population [17]. Aging has been identified as one of the most significant determinants of strained work capacity [42]. Older workers in Sweden have been more exposed to physical strain, while a shift towards more psychosocial and cognitive strain has been suggested in younger workers. One of few studies focusing work capacity found that the psychosocial work environment was an important determinant for reduced work capacity in young adults aged 21–25 years [43]. It may be that young women already face some disadvantages when they enter the labour market. In Sweden, the female-dominated labour market consists of health care, elderly and childcare, education and service sectors with generally lower salaries, less flexibility in working hours and a possibility to work from home. It is also common with emotional stress related to intense human contacts. It may be that women experiencing low mental well-being are more affected by the detrimental sides of the female-dominated labour market compared to other women. More research is needed to gain more general knowledge on capacity to work among younger women. It is important to take into consideration that a possible explanation to differences found between women and men could be related to a selection effect: men with mental health problems might be less likely to enter or remain in the labour market resulting in a hidden inequality [44] that leaves this group unrepresented in workforce statistics.

Additionally, we found an association between higher educational attainment and strained work capacity among women. This finding can be interpreted in two, not mutually exclusive ways. First, higher education may not serve as a protective factor against strained work capacity in women. Previous research suggests that educational attainment does not necessarily buffer against demanding or adverse working conditions [45]. Second, the association may reflect the persistence of occupational segregation by gender. Despite higher education levels, women are disproportionately represented in occupations characterized by unfavourable working conditions, such as emotionally demanding work [46]. Moreover, holding a managerial role appeared to be more beneficial for work capacity among men.

Results from the fully adjusted regression model (Model II) revealed a statistically significant association between mental well-being and work capacity. The direction of the association is not possible to determine in this cross-sectional study, but as discussed above, it is most likely bidirectional. There are efficient measures to relieve mental symptoms, while restoring capacity to work for return to work is more difficult [13]. It might be that more attention should be given to capacity to work at workplaces to promote work participation in persons with mental health symptoms.

For men, in addition to the significant association between low mental well-being and strained work capacity, poor health emerged as one of the strongest covariates associated with strained work capacity. This may reflect gendered help-seeking patterns, as men are less likely to seek support at an early stage, which may lead to more severe impairment. Prior studies have linked this to prevailing gender norms and stigma [47]. Workplaces could develop more open climates to reduce stigma and improve the supportive environment making it possible to share experiences of mental health problems.

In this study, we examined self-perceived mental well-being using the WHO-5 Mental Well-being Index and a newly developed instrument, the C2WI, to assess mental health-related work capacity. The two scales are supposed to measure distinct constructs. Our findings indicate that, after adjusting for factors such as sociodemographic (age and education), health (general health and long-term mental health condition), and working positions, these two constructs were strongly associated. This is on the one hand relevant since the C2WI was developed particularly to reflect capacity to work in relation to mental well-being. On the other hand, the association suggests a possible item-level overlap. One possible explanation is that the C2WI includes items that may implicitly capture physical and emotional symptoms related to mental health problems. This potential item-level overlap might contribute to the strength of the observed association and warrants consideration when interpreting the results. Further research is needed to develop and refine the C2WI or develop other instruments that may be useful in studies of mental health and work capacity in the general working population.

### Strengths and limitations

The present study is based on a cross-sectional dataset obtained through an online survey, a method that is both cost- and time-efficient [48]. This type of data helps provide a snapshot of the relationship between different variables at a given point in time, as well as mapping and identifying specific patterns and associations. However, cross-sectional data limit the ability to determine the direction of the observed associations and, as a result, the findings should be interpreted with caution. Additionally, despite adjusting for relevant covariates, we cannot rule out the possibility of residual confounding.

In relation to the recruitment process, employers were randomly selected and asked to endorse the study project by providing employee contact information. The aim was to obtain a large sample of currently employed (and working) individuals from a diverse range of occupations and regions. This proved difficult, especially in the private sector, and consequently, a web-based panel (the Norstatpanel) was used in the recruitment process. The panel is primarily used for market research and other surveys. Individuals on the panel may be biased towards better mental health and work capacity, which could lead to a selection bias, reducing the generalizability of the results. Trade unions were also used in an attempt to widen the study base. The trade unions were responsible for disseminating the survey links in the recruitment process of union members to comply with the General Data Protection Regulations. Overall, response rates were low, which is a weakness in this study. Furthermore, it is regrettable that no data are available on those who did not respond.

However, the distribution of gender and age in this study population is consistent with the national statistics of employed and currently working individuals in Sweden (i.e., the target population). Regarding the occupational categories, comparisons indicate that occupations requiring advanced university-level qualifications were more prevalent among both male and female participants. Conversely, participants in construction, manufacturing, mechanical engineering, and transport were underrepresented, as were roles requiring brief training or an introduction. Furthermore, participants may be biased towards a healthier segment of the workforce, as individuals with more severe health limitations may not be active in the workforce or are less likely to participate in a survey. This is unfortunate but common in survey research based on a working population sample [48].

In addition, participants in this study were selected through stratified random sampling, which may have introduced a potential selection bias. Another option would have been to use a general population census with a random sample. However, general population samples include unemployed individuals, people on disability pensions, and those on various types of leave. The strategy of recruiting from the workforce rather than relying on public registers resulted in a very high accuracy in terms of employment status, which is a strength of this study. We were able to recruit a substantial sample of participants from diverse occupations, ensuring comprehensive representation of the Swedish working population. Added to the limitations is a possible reporting bias as participants may selectively reveal or suppress information, adjust self-perceptions of their mental well-being, capacity to work or other variables.

Employers in public organizations were more prone to endorse the study than those in privately owned companies. This issue was addressed by using panel data. Among public organizations, the primary reason for non-endorsement was time constraints, primarily due to the ongoing impact of the COVID-19 pandemic. The main reasons for non-endorsement among privately owned companies were time constraints, a perceived lack of importance and priority regarding the study objective, and the ongoing effects of the COVID-19 pandemic.

### Practical implications

The C2WI was developed to capture the various aspects of work capacity, including how and why CMD impacts work capacity. Its items are designed to detect early, subclinical indications of reduced mental health in the working population. Future research should examine the predictive validity of the instrument in relation to work participation.

## Conclusions

This study revealed gender and age-related disparities in self-perceived mental well-being and work capacity based on the general working population. Women, particularly those aged 18–34, were more likely to report lower mental well-being and strained work capacity, compared to older women and men. Regression analyses showed that lower self-perceived mental well-being was significantly associated with more strained work capacity. To promote mental well-being and work capacity, early identification and tailored workplace interventions are recommended. Importantly, a potential limitation is the possibility of item-level overlap between the WHO-5 and C2WI instruments, which could partly explain the strength of the observed associations. Although the constructs are conceptually distinct, symptoms or experiences may impact work capacity measures. This should be considered when interpreting the results, along with the need for further validation of the C2WI.

## Supplementary Information

Supplementary Material 1.

Supplementary Material 2.

Supplementary Material 3.

Supplementary Material 4.

Supplementary Material 5.

Supplementary Material 6.

## Data Availability

The data are not publicly available because they contain information that could compromise the privacy of the study participants. Reasonable inquiries about access may be sent to the School of Public Health and Community Medicine, Institute of Medicine, Sahlgrenska Academy, University of Gothenburg, PO Box 453, SE-405 30 Gothenburg, Sweden, or by contacting: dataskydd@gu.se. The Swedish Ethical Review Authority will then be contacted for permission.

## Abbreviations

C2WI: Capacity to Work Index
CI: confidence interval
CMD: common mental disorder
WP: work participation
