# Supplementary material for: Mental well-being and work capacity: a cross-sectional study in a sample of the Swedish working population
Source: BMC Public Health. 2025 Sep 9;25:3046. doi: 10.1186/s12889-025-24015-1 (PMC12418673; doi:10.1186/s12889-025-24015-1)
Supplement: Supplementary file 1 — Supplementary Material 1. [file 12889_2025_24015_MOESM1_ESM.docx]

Additional file 1. The Swedish “Work Participation and Mental Health at Work” survey, English/Swedish version.

English version

Start of Block: Intro

Please select the language for the survey in the box above:

We will start with a few questions about you.

What is your gender?

- Woman (1)
- Man (2)
- Non-binary (3)

|  |
| --- |

What year were you born?

▼ 2004 or later (1) ... 1947 or earlier (58)

What is your level of education?
*Mark the response that best describes you.*

- Did not complete primary or lower-secondary school (1)
- Primary or lower-secondary school (2)
- Upper-secondary school or equivalent, less than 3 years (3)
- Upper-secondary school or equivalent, 3 years or more (4)
- Post-secondary education, not higher education, less than 3 years (5)
- Post-secondary education, not higher education, 3 years or more (6)
- College/university, less than 3 years (7)
- College/university, 3 years or more (8)
- Postgraduate degree (9)

Start of Block: Work life

The following questions are about your job. If you have two or more jobs, answer in terms of your primary occupation. If you are on full-time sick leave, answer the questions based on your time at work prior to going on sick leave.

In which sector do you work?

- Public sector (1)
- Private sector (2)
- Other (3)

|  |  |
| --- | --- |

What is your primary profession(s)? If you are not currently employed, state the profession that you have primarily had. 

 *A list of possible professional titles will appear when you start writing. Try to give the most detailed professional title possible. For example, write purchasing assistant instead of assistant. The list of titles is only available in Swedish. You can write in your professional title yourself if you don't know your title in Swedish or can't find it in the list.*

- (1) ________________________________________________
- *I'm not employed* (2)

Do you have a supervisory position?

- No (1)
- Yes (2)

Display This Question:

If q15 = Ja

For how many people do you have a supervisory role?

Do you work full-time or part-time?

- Full-time (1)
- Part-time (2)

Have you worked from home at any point in the last month instead of working from your regular workplace?

- Yes (1)
- No (2)

Display This Question:

If q20 = Ja

Thinking back over the last month, roughly how much of an average working week did you work at the following places?

|  | No day (1) | 1 day (2) | 2 days (3) | 3 days (4) | 4 days (5) | 5 days (6) |
| --- | --- | --- | --- | --- | --- | --- |
| From home (1) |  |  |  |  |  |  |
| At your regular workplace (2) |  |  |  |  |  |  |
| At a partner/customer (3) |  |  |  |  |  |  |
| Other: (6) |  |  |  |  |  |  |

How would you assess your current work capacity in relation to the mental and psychological demands of your work?

- Very good (1)
- Fairly good (2)
- Fair (3)
- Fairly bad (4)
- Very bad (5)

The following questions refer to your job during the past week:

|  | Not at all (1) | To a low degree (2) | To a moderate degree (3) | To a high degree (4) | *Don't know/not relevant* (5) |
| --- | --- | --- | --- | --- | --- |
| Disruptive noise has prevented me from performing my job (q26_1) |  |  |  |  |  |
| Thinking has been tough and slow (q26_2) |  |  |  |  |  |
| I have had difficulty prioritizing tasks (q26_3) |  |  |  |  |  |
| I have been able to maintain the work pace required for my work (q26_4) |  |  |  |  |  |
| I have had difficulty controlling my emotions (q26_5) |  |  |  |  |  |
| I have been sensitive to criticism from others (q26_6) |  |  |  |  |  |
| I have “put on a facade” to allow me to be at work (q26_7) |  |  |  |  |  |
| I have continued to work, even though it has caused mental or physical problems for me (q26_8) |  |  |  |  |  |
| I have had to choose to not do free-time activities to have energy to work (q26_9) |  |  |  |  |  |
| In the last week, I have gotten energy from and enjoyed my work task (q26_10) |  |  |  |  |  |
| I have had difficulty learning new work tasks (q26_11) |  |  |  |  |  |
| I have felt like a stranger at work (q26_12) |  |  |  |  |  |
| I have felt like I am closed off in a bubble, which has been a problem for me at work (q26_13) |  |  |  |  |  |
| I have avoided situations where many people physically or digitally meet because I do not have the energy to participate (q26_14) |  |  |  |  |  |
| I have felt physically weak, sore or tense, which has been an obstacle for me at work (q26_15) |  |  |  |  |  |
| I have been able to stay calm (q26_16) |  |  |  |  |  |
| I have felt wound up (q26_17) |  |  |  |  |  |

When work becomes *physically bothersome*, are you able to slow your pace or change how you work to reduce the problem?

- Yes, often (1)
- Yes, sometimes (2)
- No, rarely (3)
- No, never/almost never (4)

When work becomes *psychologically stressful*, are you able to influence your work tasks to reduce the stress?

- Yes, often (1)
- Yes, sometimes (2)
- No, rarely (3)
- No, never/almost never (4)

The following questions are about your circumstances at work.

|  | Yes, often (1) | Yes, sometimes (2) | No, rarely (3) | No, never (4) |
| --- | --- | --- | --- | --- |
| Does your job require you to work very fast? (1) |  |  |  |  |
| Does your job require you to work very hard? (2) |  |  |  |  |
| Does your job require too great a work effort? (3) |  |  |  |  |
| Do you have sufficient time for all your work tasks? (4) |  |  |  |  |
| Do conflicting demands often occur in your work? (5) |  |  |  |  |
| Do you get to learn new things at work? (6) |  |  |  |  |
| Does your job require skills? (7) |  |  |  |  |
| Does your job require creativity? (8) |  |  |  |  |
| Does your job require doing the same tasks repeatedly? (9) |  |  |  |  |
| Do you have the possibility to decide for yourself how to carry out your work? (10) |  |  |  |  |
| Do you have the possibility to decide for yourself what should be done in your work? (11) |  |  |  |  |

How true are the following statements with regard to your perspective of your workplace?

|  | Completely true (1) | Fairly true (2) | Not very true (3) | Not true at all (4) |
| --- | --- | --- | --- | --- |
| My workplace has a calm and pleasant atmosphere (1) |  |  |  |  |
| There is good collegiality at work (2) |  |  |  |  |
| My colleagues support me (3) |  |  |  |  |
| People at work understand that I may have a “bad” day (4) |  |  |  |  |
| I get along well with my supervisors (5) |  |  |  |  |
| I get along well with my co-workers (6) |  |  |  |  |

How common are conflicts at your workplace?

- Very common (1)
- Fairly common (2)
- Neither common nor uncommon (3)
- Fairly uncommon (4)
- Very uncommon (5)

Do you dislike going to work?

- Not at all (1)
- Rarely (2)
- A few days per month (3)
- One day per week (4)
- A few days per week (5)
- Every day (6)

Has bullying occurred at your workplace in the past 12 months?

- Yes, frequently (5)
- Yes, occasionally (6)
- No (7)
- *Don't know* (8)

Display This Question:

If q39 = Ja, återkommande

Or q39 = Ja, någon gång

|  |
| --- |

Have you been bullied at your workplace in the past 12 months?

- Yes, frequently (1)
- Yes, occasionally (2)
- No (3)

To what degree do you experience a conflict between your professional work and your home and household work?

- To a very high degree (1)
- To a high degree (2)
- Somewhat (3)
- To a low degree (4)
- To a very low degree (5)

Start of Block: Health

Now a few questions about your health.

In general, how would you describe your health?

- Very good (1)
- Good (2)
- Fair (3)
- Poor (4)
- Very poor (5)

Do you have a chronic illness, problem or disability?
 *You may select multiple options.*

- ⊗No (1)
- Cardiovascular disease, abnormal blood pressure (2)
- Asthma/airway symptoms/allergy (3)
- Skin disease/eczema/allergy (4)
- Symptoms/pain in muscles, joints, connective tissue  5)
- Rheumatic disease (6)
- Neurological disease (7)
- Mental health issues (8)
- Endocrine disease (such as diabetes, goiter) (9)
- Tumor (10)
- Stomach problems (11)
- Gynecological problems (12)
- Other illness, please state: (13) ________________________________________________

How often have you had the following symptoms in the past 12 months?

|  | Almost every day (1) | At some point in the week (2) | At some point in the month (3) | Almost never or never (4) |
| --- | --- | --- | --- | --- |
| Stomach problems (1) |  |  |  |  |
| Heart palpitations (2) |  |  |  |  |
| Breathing difficulties (3) |  |  |  |  |
| Fatigue (4) |  |  |  |  |
| Dizziness (5) |  |  |  |  |
| Headache (6) |  |  |  |  |
| Chest pain (7) |  |  |  |  |
| Back pain, sciatica (8) |  |  |  |  |
| Neck and/or shoulder pain (9) |  |  |  |  |
| Difficulty concentrating (12) |  |  |  |  |

How have you felt in the past two weeks?

 *Please indicate for each of the five statements which is closest to how you have been feeling over the last two weeks.*

|  | All the time (6) | Most of the time (5) | More than half of the time (4) | Less than half of the time (3) | Some of the time (2) | At no time (1) |
| --- | --- | --- | --- | --- | --- | --- |
| I have felt cheerful and in good spirits (1) |  |  |  |  |  |  |
| I have felt calm and relaxed (2) |  |  |  |  |  |  |
| I have felt active and vigorous (3) |  |  |  |  |  |  |
| I woke up feeling fresh and rested (4) |  |  |  |  |  |  |
| My daily life has been filled with things that interest me (10) |  |  |  |  |  |  |

Are you currently in contact with the health care system due to mental health problems?

- Yes (1)
- No (2)

To what extent have you taken the following prescription medications in the past month?

|  | Essentially every day (1) | Several times per week (2) | A few times per month (3) | Never (4) |
| --- | --- | --- | --- | --- |
| Antidepressants (1) |  |  |  |  |
| Anti-anxiety medication (2) |  |  |  |  |
| Sleeping medication (3) |  |  |  |  |

In the past month, how often have you...

|  | Essentially every day (1) | Several times per week (2) | A few times per month (3) | Never (4) |
| --- | --- | --- | --- | --- |
| Woken up feeling well-rested (1) |  |  |  |  |
| Had difficulty with sleep (such as difficulty falling asleep, waking up repeatedly, waking too early) (2) |  |  |  |  |
| Felt tired at work or in your free time (3) |  |  |  |  |

Start of Block: Sickness absence

Are you currently on sick leave?

- No (1)
- Yes, part time (2)
- Yes, full time (3)

Display This Question:

If q60 = Ja, deltid

Or q60 = Ja, heltid

How long have you been on sick leave?

 *Include all days of the week, Saturday and Sunday as well.*

- 1 – 7 days (1)
- 8 – 14 days (2)
- 15 days - 12 months (3)

Display This Question:

If q60 = Nej

Have you been on sick leave at any point in the past 12 months?

- No (1)
- Yes (2)

Display This Question:

If q62 = Ja

In total, how much time have you been on sick leave in the past 12 months?

 *Include all days of the week, Saturday and Sunday as well.*

- 1 – 7 days (1)
- 8 – 14 days (2)
- 15 days - 12 months (3)

Are you currently receiving sickness benefit or activity compensation?

- No (1)
- Yes, part time (2)
- Yes, full time (3)

To answer one of the questions in the study we follow up on sick leave that exceeds 14 days in the Swedish Social Insurance Agency's register. Follow-up time is a maximum of 12 months from the time the questionnaire is answered.

 Do you agree to a follow up of register data?

- Yes, I agree (1)
- No, I don't agree (2)

Start of Block: Family life

The following questions are about your family life.

Do you have any children living at home?

 *Include children who live at your home at least part time, whether they are your biological children, adopted children, foster children or your partner’s children.*

- Yes (1)
- No (2)

Display This Question:

If q70 = Ja

q71 How many children live at home in the following age range (years)?

- age 0-5 (1) ________________________________________________
- age 6-12 (2) ________________________________________________
- age 13-17 (3) ________________________________________________
- age 18 or older (4) ________________________________________________

Start of Block: Life events

The last question is about physical activity and exercise.

How much have you moved and physically exerted yourself in your free time in the past 12 months?

 *If your activity level has varied significantly from summer to winter, for example, try to give an average.*

- Sedentary free time (you are almost completely physically inactive: you spend your free time reading, watching TV and movies, using the computer, or engaging in other sedentary activities) (1)
- Some physical activity in your free time, at least 4 hours per week (you ride a bike or walk to work for example, take walks or ski with your family, gardening, fishing, table tennis, bowling etc.) (2)
- Regular moderate physical activity and exercise, at least 2 to 3 hours per week (such as heavy gardening work, running, swimming, fitness classes, tennis, badminton or similar activities) (3)
- Regular intense exercise and competitive sports (high-intensity activity) (running, orienteering, skiing, swimming, football, handball etc. several times per week) (4)

Start of Block: outro

**Thank you for your responses!**

 If you have questions about the study or want to discuss something, you are welcome to contact us. Please contact Professor Gunnel Hensing at the School of Public Health and Community Medicine at the University of Gothenburg by email:

 If you have comments on this survey or other things, you are welcome to write them here:

________________________________________________________________

________________________________________________________________

________________________________________________________________

EU37 ADAPT/Capacity 2 Work – Swedish version

Start of Block: Intro/Bakgrund

Vänligen välj språk för enkäten i rutan ovan /

Först kommer några frågor om dig själv.

Vilket kön har du?

- Kvinna (1)
- Man (2)
- Icke-binär (3)

|  |
| --- |

Vilket år är du född?

▼ 2004 eller senare (1) ... 1947 eller tidigare (58)

Vilken skolutbildning har du?
 *Markera det svar som bäst stämmer in på dig.*

- Ej fullgjort grundskola (1)
- Grundskola (2)
- Gymnasium eller motsvarande, kortare än 3 år (3)
- Gymnasium eller motsvarande, 3 år eller längre (4)
- Eftergymnasial utbildning, ej högskola, kortare än 3 år (5)
- Eftergymnasial utbildning, ej högskola, 3 år eller längre (6)
- Högskola/universitet, kortare än 3 år (7)
- Högskola/universitet, 3 år eller längre (8)
- Examen från forskarutbildning (9)

Start of Block: Arbetsliv

Nu följer några frågor om ditt arbete. Har du två eller flera arbeten, utgå från ditt huvudsakliga arbete. Om du är heltidssjukskriven besvarar du frågorna utifrån hur det var på ditt arbete innan du blev sjukskriven.

Inom vilken sektor arbetar du?

- Offentlig sektor (1)
- Privat sektor (2)
- Övrig (3)

|  |  |
| --- | --- |

Vilket är/var ditt huvudsakliga yrke? Om du inte är yrkesverksam för närvarande gäller frågan ditt senaste yrke.


*När du börjar skriva in ditt yrke kommer du få upp en lista med passande förslag. Välj det yrke i listan som ligger närmast dina arbetsuppgifter. Skriv annars vad du själv skulle kalla det.*

- (1) ________________________________________________
- *Jag har ingen anställning* (2)

Har du en arbetsledande befattning?

- Nej (1)
- Ja (2)

Display This Question:

If q15 = Ja

För hur många personer har du en arbetsledande funktion?

Arbetar du heltid eller deltid?

- Heltid (1)
- Deltid (2)

Har du någon gång under den senaste månaden arbetat hemifrån istället för därifrån du vanligtvis arbetar?

- Ja (1)
- Nej (2)

Display This Question:

If q20 = Ja

Tänk tillbaka på den senaste månaden, ungefär hur stor del av en genomsnittlig arbetsvecka har du arbetat på följande platser?

|  | Ingen dag (1) | 1 dag (2) | 2 dagar (3) | 3 dagar (4) | 4 dagar (5) | 5 dagar (6) |
| --- | --- | --- | --- | --- | --- | --- |
| Hemma (1) |  |  |  |  |  |  |
| På ordinarie arbetsplats (2) |  |  |  |  |  |  |
| Hos samarbetspartner/kund (3) |  |  |  |  |  |  |
| Annat: (6) |  |  |  |  |  |  |

Hur bedömer du din nuvarande arbetsförmåga vara i förhållande till de mentala och psykiska krav arbetet ställer?

- Mycket god (1)
- Ganska god (2)
- Någorlunda (3)
- Ganska dålig (4)
- Mycket dålig (5)

Följande frågor handlar om hur du har haft det i ditt arbete under den senaste veckan:

|  | Inte alls (1) | I låg grad (2) | I måttlig grad (3) | I hög grad (4) | *Vet ej / ej relevant* (5) |
| --- | --- | --- | --- | --- | --- |
| Störande ljud har hindrat mig i mitt arbete (q26_1) |  |  |  |  |  |
| Att tänka har varit segt och trögt (q26_2) |  |  |  |  |  |
| Jag har haft svårt att prioritera bland mina arbetsuppgifter (q26_3) |  |  |  |  |  |
| Jag har kunnat hålla det arbetstempo som har krävts i mitt arbete (q26_4) |  |  |  |  |  |
| Jag har haft svårt att kontrollera mina känslor (q26_5) |  |  |  |  |  |
| Jag har varit känslig för kritik från människor jag mött (q26_6) |  |  |  |  |  |
| Jag har ”satt på mig en fasad” för att kunna vara på jobbet (q26_7) |  |  |  |  |  |
| Jag har fortsatt att arbeta trots att jag har fått psykiska eller kroppsliga besvär av det (q26_8) |  |  |  |  |  |
| Jag har behövt välja bort saker på min fritid för att orka arbeta (q26_9) |  |  |  |  |  |
| Senaste veckan har jag fått energi och arbetsglädje av mina arbetsuppgifter (q26_10) |  |  |  |  |  |
| Jag har haft svårt att lära mig nya arbetsuppgifter (q26_11) |  |  |  |  |  |
| Jag har känt mig som en främling på jobbet (q26_12) |  |  |  |  |  |
| Jag har känt mig som instängd i en bubbla vilket har hindrat mig i mitt arbete (q26_13) |  |  |  |  |  |
| Jag har undvikit tillfällen där många träffas, fysiskt eller digitalt, för att jag inte orkat delta (q29_14) |  |  |  |  |  |
| Jag har känt mig svag, öm eller spänd i min kropp vilket har hindrat mig i mitt arbete (q26_15) |  |  |  |  |  |
| Jag har kunnat behålla mitt lugn (q26_16) |  |  |  |  |  |
| Jag har varit uppvarvad (q26_17) |  |  |  |  |  |

När arbetet blir *kroppsligt besvärande*, har du då möjlighet att dra ner på takten eller arbeta annorlunda, så att besvären minskar?

- Ja, ofta (1)
- Ja, ibland (2)
- Nej, sällan (3)
- Nej, aldrig/nästan aldrig (4)

När arbetet blir *psykiskt påfrestande*, har du då möjlighet att påverka det du gör, så att påfrestningen minskar?

- Ja, ofta (1)
- Ja, ibland (2)
- Nej, sällan (3)
- Nej, aldrig/nästan aldrig (4)

Nu följer några frågor om hur du har det på ditt arbete.

|  | Ja, ofta (1) | Ja, ibland (2) | Nej, sällan (3) | Nej, aldrig (4) |
| --- | --- | --- | --- | --- |
| Kräver ditt arbete att du arbetar mycket fort? (1) |  |  |  |  |
| Kräver ditt arbete att du arbetar mycket hårt? (2) |  |  |  |  |
| Kräver ditt arbete en för stor arbetsinsats? (3) |  |  |  |  |
| Har du tillräckligt med tid för att hinna med arbetsuppgifterna? (4) |  |  |  |  |
| Förekommer det motstridiga krav ditt arbete? (5) |  |  |  |  |
| Får du lära dig nya saker i ditt arbete? (6) |  |  |  |  |
| Kräver ditt arbete skicklighet? (7) |  |  |  |  |
| Kräver ditt arbete påhittighet? (8) |  |  |  |  |
| Innebär ditt arbete att man gör samma sak om och om igen? (9) |  |  |  |  |
| Har du frihet att bestämma hur ditt arbete ska utföras? (10) |  |  |  |  |
| Har du frihet att bestämma vad som ska utföras i ditt arbete? (11) |  |  |  |  |

Hur väl stämmer följande in på dig när det gäller din syn på din arbetsplats?

|  | Stämmer helt och hållet (1) | Stämmer ganska bra (2) | Stämmer inte särskilt bra (3) | Stämmer inte alls (4) |
| --- | --- | --- | --- | --- |
| Det är en lugn och behaglig stämning på min arbetsplats (1) |  |  |  |  |
| Det är en god sammanhållning (2) |  |  |  |  |
| Mina arbetskamrater ställer upp för mig (3) |  |  |  |  |
| Man har förståelse för att jag kan ha en dålig dag (4) |  |  |  |  |
| Jag kommer bra överens med mina överordnade (5) |  |  |  |  |
| Jag trivs bra med mina arbetskamrater (6) |  |  |  |  |

Hur vanligt är det att det förekommer konflikter på din arbetsplats?

- Mycket vanligt (1)
- Ganska vanligt (2)
- Varken vanligt eller ovanligt (3)
- Ganska ovanligt (4)
- Mycket ovanligt (5)

Händer det att du känner olust när du går till arbetet?

- Inte alls (1)
- Sällan (2)
- Ett par dagar per månad (3)
- En dag per vecka (4)
- Ett par dagar per vecka (5)
- Varje dag (6)

Har det förekommit mobbning på din arbetsplats under de senaste 12 månaderna?

- Ja, återkommande (5)
- Ja, någon gång (6)
- Nej (7)
- *Vet ej* (8)

Display This Question:

If q39 = Ja, återkommande

Or q39 = Ja, någon gång

|  |
| --- |

Har du själv varit utsatt för mobbning på din arbetsplats under de senaste 12 månaderna?

- Ja, återkommande (1)
- Ja, någon gång (2)
- Nej (3)

I vilken grad upplever du en konflikt mellan yrkesarbetet och uppgifter i hem och hushåll?

- I mycket hög grad (1)
- I hög grad (2)
- Delvis (3)
- I låg grad (4)
- I mycket låg grad (5)

Start of Block: Hälsa

Nu följer några frågor om din hälsa.

I allmänhet, hur skulle du beskriva din hälsa?

- Mycket bra (1)
- Bra (2)
- Någorlunda (3)
- Dålig (4)
- Mycket dålig (5)

|  |
| --- |

Har du någon varaktig sjukdom, besvär eller handikapp?
 *Flera alternativ kan anges.*

- ⊗Nej (1)
- Hjärt-kärlsjukdom, onormalt blodtryck (2)
- Astma/luftrörsbesvär/allergi (3)
- Hudsjukdom/eksem/allergi (4)
- Symptom/smärta i muskler, leder, bindväv (5)
- Reumatisk sjukdom (6)
- Neurologisk sjukdom (7)
- Psykiska besvär (8)
- Endokrinologisk sjukdom (t ex diabetes, struma) (9)
- Tumörsjukdom (10)
- Magbesvär (11)
- Gynekologiska besvär (12)
- Annan sjukdom, ange vilken: (13) ________________________________________________

|  |
| --- |

Hur ofta har du haft följande symptom under de senaste 12 månaderna?

|  | Nästan varje dygn (1) | Någon gång i veckan (2) | Någon gång i månaden (3) | Nästan aldrig eller aldrig (4) |
| --- | --- | --- | --- | --- |
| Magbesvär (1) |  |  |  |  |
| Hjärtklappning (2) |  |  |  |  |
| Andningsbesvär (3) |  |  |  |  |
| Trötthet (4) |  |  |  |  |
| Yrsel (5) |  |  |  |  |
| Huvudvärk (6) |  |  |  |  |
| Smärtor i bröstet (7) |  |  |  |  |
| Ryggsmärtor, ryggvärk, ischias (8) |  |  |  |  |
| Värk i nacke och/eller skuldervärk (9) |  |  |  |  |
| Svårt att koncentrera mig (12) |  |  |  |  |

|  |
| --- |

Hur har du känt dig de senaste veckorna?

*Var vänlig markera för varje påstående vad som är närmast hur du har känt under de senaste 2 veckorna.*

|  | Hela tiden (6) | Mestadels (5) | Mer än halva tiden (4) | Mindre än halva tiden (3) | Ibland (2) | Aldrig (1) |
| --- | --- | --- | --- | --- | --- | --- |
| Jag har känt mig glad och på gott humör (1) |  |  |  |  |  |  |
| Jag har känt mig lugn och avslappnad (2) |  |  |  |  |  |  |
| Jag har känt mig aktiv och kraftfull (3) |  |  |  |  |  |  |
| Jag har känt mig pigg och utvilad när jag vaknat (4) |  |  |  |  |  |  |
| Mitt vardagsliv har varit fyllt av sådant som intresserar mig (10) |  |  |  |  |  |  |

|  |
| --- |

Har du för närvarande kontakt med hälso- och sjukvården för psykiska problem?

- Ja (1)
- Nej (2)

I vilken utsträckning har du använt följande receptbelagda mediciner den senaste månaden?

|  | I stort sett varje dag (1) | Flera gånger per vecka (2) | Någon/några gånger per månad (3) | Aldrig (4) |
| --- | --- | --- | --- | --- |
| Läkemedel mot depression (1) |  |  |  |  |
| Läkemedel mot ångest (2) |  |  |  |  |
| Läkemedel mot sömnbesvär (3) |  |  |  |  |

Hur ofta har du den senaste månaden...

|  | I stort sett varje dag (1) | Flera gånger per vecka (2) | Någon/några gånger per månad (3) | Aldrig (4) |
| --- | --- | --- | --- | --- |
| Vaknat utsövd (1) |  |  |  |  |
| Haft besvär med sömnen (t ex haft svårt att somna, upprepade uppvaknanden, för tidigt uppvaknande) (2) |  |  |  |  |
| Varit sömnig under arbete eller fritid (3) |  |  |  |  |

Start of Block: Sjukskrivning

Är du sjukskriven just nu?

- Nej (1)
- Ja, deltid (2)
- Ja, heltid (3)

Display This Question:

If q60 = Ja, deltid

Or q60 = Ja, heltid

Hur länge har du varit sjukskriven?

 *Räkna med veckans alla dagar alltså även lördag och söndag.*

- 1 – 7 dagar (1)
- 8 – 14 dagar (2)
- 15 dagar - 12 månader (3)

Display This Question:

If q60 = Nej

Har du varit sjukskriven vid något tillfälle under de senaste 12 månaderna?

- Nej (1)
- Ja (2)

Display This Question:

If q62 = Ja

Hur lång tid har du sammanlagt varit sjukskriven under de senaste 12 månaderna?

 *Räkna med veckans alla dagar alltså även lördag och söndag.*

- 1 – 7 dagar (1)
- 8 – 14 dagar (2)
- 15 dagar - 12 månader (3)

Har du sjuk- eller aktivitetsersättning just nu?

- Nej (1)
- Ja, på deltid (2)
- Ja, på heltid (3)

För att besvara en av frågeställningarna i studien behöver vi följa upp sjukfrånvaro som överstiger 14 dagar i Försäkringskassans register. Uppföljningstiden är maximalt 12 månader från det att enkäten besvaras.

Godkänner du att vi följer upp registerdata?

- Ja, jag godkänner (1)
- Nej, jag godkänner inte (2)

Start of Block: Familjeförhållanden

Nu följer några frågor om dina familjeförhållanden.

Har du några barn som bor hemma?
 *Räkna med de barn som bor minst halva tiden hos dig oavsett om det är dina egna, familjehemsbarn eller maka/make/sambos barn.*

- Ja (1)
- Nej (2)

Display This Question:

If q70 = Ja

Hur många barn bor hemma i följande åldersintervall (år)?

- 0-5 år (1) ________________________________________________
- 6-12 år (2) ________________________________________________
- 13-17 år (3) ________________________________________________
- 18 år eller äldre (4) ________________________________________________

Start of Block: Livshändelser

Slutligen följer en fråga om fysisk aktivitet och motion.

Hur mycket har du rört på dig och ansträngt dig kroppsligt på fritiden under de senaste 12 månaderna?

 *Om din aktivitet varierat mycket mellan t ex sommar och vinter, så försök att ta ett genomsnitt.*

- Stillasittande fritid (du är nästan helt fysisk inaktiv: läser, ser på TV och film, använder dator eller har annan stillasittande sysselsättning på fritiden) (1)
- Någon fysisk aktivitet på fritiden under minst 4 timmar per vecka (du cyklar eller promenerar exempelvis till arbetet, promenerar eller åker skidor med familjen, trädgårdsarbete, fiske, bordtennis, bowling etc) (2)
- Regelbunden måttlig fysisk aktivitet och träning under minst 2 till 3 timmar per vecka (du ägnar dig åt t.ex. tungt trädgårdsarbete, löpning, simning, motionsgymnastik, tennis, badminton eller liknande aktiviteter) (3)
- Regelbunden hård träning och tävlingsidrott (aktivitet med hög intensitet) (du ägnar dig åt löpning, orientering, skidåkning, simning, fotboll, handboll etc. flera gånger i veckan) (4)

Start of Block: Outro

q78 **Stort tack för dina svar!** Har du frågor kring studien eller vill diskutera något är du välkommen att kontakta oss. Ta då kontakt med professor Gunnel Hensing vid Avdelningen för samhällsmedicin och folkhälsa vid Göteborgs universitet via följande e-postadress:

Har du synpunkter på den här undersökningen eller övriga kommentarer får du gärna lämna dem här:

________________________________________________________________

________________________________________________________________

________________________________________________________________

________________________________________________________________

________________________________________________________________
