# Supplementary material for: Mental well-being and work capacity: a cross-sectional study in a sample of the Swedish working population
Source: BMC Public Health. 2025 Sep 9;25:3046. doi: 10.1186/s12889-025-24015-1 (PMC12418673; doi:10.1186/s12889-025-24015-1)
Supplement: Supplementary file 2 — Supplementary Material 2. [file 12889_2025_24015_MOESM2_ESM.docx]

**Additional file 2**. The Capacity to Work instrument (C2WI), item-by-item responses (response option 1-4), *n*=8462

|  | Not at all (1) | | To a low degree (2) | | | | To a moderate degree (3) | | | | | To a high degree (4) | | | | |  |
| --- | --- | --- | --- | --- | --- | --- | --- | --- | --- | --- | --- | --- | --- | --- | --- | --- | --- |
| *The following statements refer to your job during the past week:* | n | (%) | | n | (%) | | | n | | (%) | | | n | | (%) | |  |
| Disruptive noise prevented me from performing my job. | 3532 | (42) | | 2530 | | (30) | | | 1729 | | (20) | | | 671 | | (8) | |
| Thinking has been tough and slow. | 2477 | (29) | | 2854 | | (34) | | | 2211 | | (26) | | | 920 | | (11) | |
| I have had difficulty prioritizing tasks. | 3066 | (36) | | 2709 | | (32) | | | 1909 | | (23) | | | 778 | | (9) | |
| I have had difficulty controlling my emotions. | 4447 | (53) | | 2194 | | (26) | | | 1263 | | (15) | | | 558 | | (7) | |
| I have been sensitive to criticism from others. | 3614 | (43) | | 2675 | | (32) | | | 1526 | | (18) | | | 647 | | (8) | |
| I have “put on a facade” to allow me to be at work. | 4110 | (49) | | 1902 | | (22) | | | 1401 | | (17) | | | 1049 | | (12) | |
| I have continued to work, even though it has caused mental or physical problems for me. | 4305 | (51) | | 1575 | | (19) | | | 1267 | | (15) | | | 1315 | | (16) | |
| I have had to choose to not do free-time activities to have energy to work. | 3454 | (41) | | 1888 | | (22) | | | 1595 | | (19) | | | 1525 | | (18) | |
| I have had difficulty learning new work tasks. | 5102 | (60) | | 2197 | | (26) | | | 917 | | (11) | | | 246 | | (3) | |
| I have felt like a stranger at work. | 5897 | (70) | | 1465 | | (17) | | | 801 | | (9) | | | 299 | | (4) | |
| I have felt like I am closed off in “a bubble”, which has been a problem for me at work. | 5842 | (69) | | 1572 | | (19) | | | 815 | | (10) | | | 233 | | (3) | |
| I have avoided situations where many people physically or digitally meet because I do not have the energy to participate. | 4755 | (56) | | 1737 | | (21) | | | 1153 | | (14) | | | 817 | | (10) | |
| I have felt physically weak, sore, or tense, which has been an obstacle for me at work. | 4679 | (55) | | 2080 | | (25) | | | 1168 | | (14) | | | 535 | | (6) | |
| I have felt wound up. | 1993 | (24) | | 2844 | | (34) | | | 2580 | | (30) | | | 1045 | | (12) | |
