# Supplementary material for: Mental well-being and work capacity: a cross-sectional study in a sample of the Swedish working population
Source: BMC Public Health. 2025 Sep 9;25:3046. doi: 10.1186/s12889-025-24015-1 (PMC12418673; doi:10.1186/s12889-025-24015-1)
Supplement: Supplementary file 3 — Supplementary Material 3. [file 12889_2025_24015_MOESM3_ESM.docx]

**Additional file 3**. The distribution of the WHO–5 Mental Well-being Index in a cohort based on the Swedish “Work Participation and Mental Health at Work” (ADAPT) research project, 2021–2022, stratified by gender, (*n*=8462).

|  | Women (*n*=4905) (58%) | | | | | | Men (*n*=3557) (42%) | | | | | | | | | | |
| --- | --- | --- | --- | --- | --- | --- | --- | --- | --- | --- | --- | --- | --- | --- | --- | --- | --- |
|  | WHO-5 (score ≤44) | | | WHO-5 (score ≥45) | | | WHO-5 (score ≤44) | | | | | | WHO-5 (score ≥45) | | | | |
|  | *n* | % |  | *n* | % |  | *n* | | % |  | | | *n* | | % |  | |
| *Age groups* |  | | | | | | | | | | | | | | | | |
| 18–34 years | 403 | 35 |  | 747 | 65 |  | 165 | 21 | | |  | 630 | | 79 | | |  |
| 35–54 years | 797 | 32 |  | 1694 | 68 |  | 408 | 23 | | |  | 1355 | | 77 | | |  |
| 55–74 years | 298 | 24 |  | 926 | 76 |  | 160 | 16 | | |  | 827 | | 84 | | |  |
| *Level of education* |  | | | | | | | | | | | | | | | | |
| University or higher (≥16 years) | 800 | 30 |  | 1852 | 70 |  | 304 | 22 | | |  | 1104 | | 80 | | |  |
| Post secondary (13–15 years) | 134 | 29 |  | 329 | 71 |  | 83 | 21 | | |  | 313 | | 81 | | |  |
| Upper secondary (10–12 years) | 416 | 32 |  | 896 | 68 |  | 248 | 20 | | |  | 997 | | 80 | | |  |
| Lower secondary or less (≤9 years) | 148 | 34 |  | 291 | 66 |  | 98 | 20 | | |  | 400 | | 80 | | |  |
| *Occupational Classification* |  | | | | | | | | | | | | | | | | |
| Non-manual, high-skilled | 917 | 29 |  | 2282 | 71 |  | 435 | 19 | | |  | 1848 | | 81 | | |  |
| Non-manual, low-skilled | 90 | 33 |  | 179 | 67 |  | 19 | 27 | | |  | 51 | | 73 | | |  |
| Manual, high-skilled | 81 | 31 |  | 181 | 69 |  | 126 | 19 | | |  | 527 | | 81 | | |  |
| Manual, low-skilled | 402 | 36 |  | 708 | 64 |  | 144 | 30 | | |  | 337 | | 70 | | |  |
| *Managerial position* |  | | | | | | | | | | | | | | | | |
| Yes | 214 | 23 |  | 702 | 77 |  | 174 | 18 | | |  | 804 | | 82 | | |  |
| No | 1283 | 32 |  | 2667 | 68 |  | 559 | 22 | | |  | 2009 | | 78 | | |  |
| *Working full-time* |  | | | | | | | | | | | | | | | | |
| Yes | 1103 | 29 |  | 2689 | 71 |  | 642 | 20 | | |  | 2607 | | 80 | | |  |
| No | 395 | 37 |  | 678 | 63 |  | 89 | 30 | | |  | 204 | | 70 | | |  |
| *SF-36 General Health* |  | | | | | | | | | | | | | | | | |
| Good/very good general health | 466 | 15 |  | 2650 | 85 |  | 236 | 9 | | |  | 2328 | | 91 | | |  |
| Moderate general health | 699 | 50 |  | 697 | 50 |  | 259 | 33 | | |  | 519 | | 67 | | |  |
| Poor/very poor general health | 288 | 79 |  | 76 | 21 |  | 135 | 66 | | |  | 71 | | 34 | | |  |
| *Long-term health conditions* |  | | | | | | | | | | | | | | | | |
| No | 322 | 19 |  | 1394 | 81 |  | 216 | 13 | | |  | 1456 | | 87 | | |  |
| Yes, mental health conditions | 135 | 56 |  | 106 | 44 |  | 63 | 51 | | |  | 61 | | 49 | | |  |
| Yes, mental health conditions and other | 446 | 62 |  | 275 | 38 |  | 140 | 63 | | |  | 81 | | 37 | | |  |
| Other than mental health conditions | 586 | 27 |  | 1563 | 73 |  | 313 | 21 | | |  | 1208 | | 79 | | |  |
| *Work sector* |  | | | | | | | | | | | | | | | | |
| Public sector | 1035 | 32 |  | 2240 | 68 |  | 302 | 22 | | |  | 1044 | | 78 | | |  |
| Private sector | 436 | 28 |  | 1096 | 72 |  | 427 | 20 | | |  | 1754 | | 80 | | |  |
