# Supplementary material for: Mental well-being and work capacity: a cross-sectional study in a sample of the Swedish working population
Source: BMC Public Health. 2025 Sep 9;25:3046. doi: 10.1186/s12889-025-24015-1 (PMC12418673; doi:10.1186/s12889-025-24015-1)
Supplement: Supplementary file 4 — Supplementary Material 4. [file 12889_2025_24015_MOESM4_ESM.docx]

**Additional file 4**.The distribution of capacity to work (C2WI) in a cohort based on the Swedish “Work Participation and Mental Health at Work” (ADAPT) research project, 2021–2022, stratified by gender (*n*=8462).

|  | | | | | | | | | | | | | | | |
| --- | --- | --- | --- | --- | --- | --- | --- | --- | --- | --- | --- | --- | --- | --- | --- |
|  | Women (*n*=4905) (58%) | | | | | | | | | Men (*n*=3557) (42%) | | | | | |
|  | C2WI (score ≥33) | | | | | | C2WI (score ≤32) | | | C2WI (score ≥33) | | | C2WI (score ≤32) | | |
|  | *n* | | | | % |  | *n* | % |  | *n* | % |  | *n* | % |  |
| *Age groups* | | |  | | | | | | | | | | | | |
| 18–34 years | 432 | | | | 37 |  | 730 | 63 |  | 164 | 21 |  | 633 | 79 |  |
| 35–54 years | 754 | | | | 30 |  | 1756 | 70 |  | 335 | 19 |  | 1433 | 81 |  |
| 55–74 years | 280 | | | | 23 |  | 951 | 77 |  | 133 | 13 |  | 857 | 87 |  |
| *Level of education* |  | | | |  |  |  |  |  |  |  |  |  |  |  |
| University or higher (≥16 years) | 819 | | | | 31 |  | 1851 | 69 |  | 241 | 17 |  | 1172 | 83 |  |
| Post secondary (13–15 years) | 139 | | | | 30 |  | 326 | 70 |  | 82 | 21 |  | 317 | 79 |  |
| Upper secondary (10–12 years) | 391 | | | | 30 |  | 930 | 70 |  | 222 | 18 |  | 1024 | 82 |  |
| Lower secondary or less (≤9 years) | 117 | | | | 26 |  | 329 | 74 |  | 88 | 18 |  | 411 | 82 |  |
| *Occupational Classification* |  | | | |  |  |  |  |  |  |  |  |  |  |  |
| Non-manual, high-skilled | 913 | | | | 28 |  | 2305 | 72 |  | 364 | 16 |  | 1926 | 84 |  |
| Non-manual, low-skilled | 75 | | | | 28 |  | 194 | 72 |  | 12 | 17 |  | 58 | 83 |  |
| Manual, high-skilled | 68 | | | | 26 |  | 194 | 74 |  | 110 | 17 |  | 546 | 83 |  |
| Manual, low-skilled | 399 | | | | 35 |  | 730 | 65 |  | 132 | 27 |  | 349 | 73 |  |
| *Managerial position* |  | | | |  |  |  |  |  |  |  |  |  |  |  |
| Yes | 231 | | | | 25 |  | 694 | 75 |  | 169 | 17 |  | 810 | 83 |  |
| No | 1234 | | | | 33 |  | 2475 | 67 |  | 464 | 18 |  | 2113 | 82 |  |
| *Working full-time* |  | | | |  |  |  |  |  |  |  |  |  |  |  |
| Yes | 1053 | | | | 28 |  | 2764 | 72 |  | 550 | 17 |  | 2707 | 83 |  |
| No | 413 | | | | 38 |  | 673 | 62 |  | 80 | 27 |  | 215 | 73 |  |
| *SF-36 General Health* |  | | | |  |  |  |  |  |  |  |  |  |  |  |
| Good/very good general health | 466 | | | | 15 |  | 2650 | 85 |  | 236 | 9 |  | 2328 | 91 |  |
| Moderate general health | 699 | | | | 50 |  | 697 | 50 |  | 259 | 33 |  | 519 | 67 |  |
| Poor/very poor general health | 288 | | | | 79 |  | 76 | 21 |  | 135 | 65 |  | 71 | 35 |  |
| *WHO-5 Mental Well-being Index*  (range, 0–100) |  | | | |  |  |  |  |  |  |  |  |  |  |  |
| Mental well-being (≥45) | 475 | | | | 14 |  | 2894 | 86 |  | 248 | 9 |  | 2566 | 91 |  |
| Mental well-being (≤44) | 972 | | | | 65 |  | 526 | 35 |  | 382 | 52 |  | 351 | 48 |  |
| *Long-term health conditions* | | | |  |  |  |  |  |  |  |  |  |  |  |  |
| No | | 321 | | | 19 |  | 1398 | 81 |  | 188 | 11 |  | 1484 | 89 |  |
| Yes, mental health conditions | | 142 | | | 59 |  | 100 | 41 |  | 70 | 57 |  | 54 | 43 |  |
| Yes, mental health conditions and others | | 453 | | | 63 |  | 269 | 37 |  | 126 | 57 |  | 95 | 43 |  |
| Other than mental health conditions | | 524 | | | 24 |  | 1627 | 76 |  | 246 | 16 |  | 1276 | 84 |  |
| *Work sector* | | | |  |  |  |  |  |  |  |  |  |  |  |  |
| Public sector | | | | 1044 | 32 |  | 2261 | 68 |  | 267 | 20 |  | 1088 | 80 |  |
| Private sector | | | | 398 | 26 |  | 1139 | 74 |  | 357 | 16 |  | 1825 | 84 |  |
