# Supplementary material for: Mental well-being and work capacity: a cross-sectional study in a sample of the Swedish working population
Source: BMC Public Health. 2025 Sep 9;25:3046. doi: 10.1186/s12889-025-24015-1 (PMC12418673; doi:10.1186/s12889-025-24015-1)
Supplement: Supplementary file 5 — Supplementary Material 5. [file 12889_2025_24015_MOESM5_ESM.docx]

| **Additional file 5**. Univariable linear regression analysis of capacity to work and associated factors in a cohort based on the Swedish “Work Participation and Mental Health at Work” (ADAPT) research project, 2021 – 2022, stratified by gender (women: *n*=4905).   \| **Variables** \| **B** \| \| **95% CI** \| \| --- \| --- \| --- \| --- \| \| *WHO-5 Mental Well-being Index* (range, 0–100) \| –0.322 \| \| (–0.332;-0.313) \| \| *Age groups* \|  \| \|  \| \| 18 – 34 years (Ref.) \|  \| \|  \| \| 35 – 54 years \| –2.148 \| \| (–2.808;–1.487) \| \| 55 – 74 years \| –4.348 \| \| (–5.109;–3.587) \| \| *Education level* \|  \| \|  \| \| University or higher (≥16 years) (Ref.) \|  \| \|  \| \| Post secondary (13–15 years) \| 1.444 \| \| (0.481;2.408) \| \| Upper secondary (10–12 years) \| 1.304 \| \| (0.272;2.335) \| \| Lower secondary or less (≤9 years) \| 0.818 \| \| (–0.430;2.066) \| \| *Occupational classification* \|  \| \|  \| \| Non-manual, high-skilled (Ref.) \|  \| \|  \| \| Non-manual, low-skilled \| –0.832 \| \| (–2.025;0.361) \| \| Manual, high-skilled \| –0.653 \| \| (–1.861;0.554) \| \| Manual, low-skilled \| 1.518 \| \| (0.868;2.168) \| \| *Managerial position* \|  \| \|  \| \| Yes (Ref.) \|  \| \|  \| \| No \| 1.464 \| \| (0.777;2.150) \| \| *Working time* \|  \| \|  \| \| Full-time (Ref.) \|  \| \|  \| \| Part–time \| 2.021 \| \| (1.376;2.667) \| \| *SF-36 General Health* \|  \| \|  \| \| Good/very good (Ref.) \|  \| \|  \| \| Moderate \| 8.533 \| \| (8.022;9.044) \| \| Poor/Very poor \| 16.019 \| \| (15.140;16.897) \| \| *Long–term mental health condition* \|  \| \|  \| \| No (Ref.) \|  \| \|  \| \| Yes \| 9.548 \| \| (8.925;10.170) \| \| *Work sector* \|  \| \|  \| \| Private (Ref.) \|  \| \|  \| \| Public \| 1.107 \| \| (0.527;1.687) \| \| Higher scores on the outcome indicate a more strained work capacity.  Abbreviations: B, Unstandardized Coefficient. CI, Confidence Interval. Ref., Reference category. \| \|  \| \| \| |
| --- | --- | --- | --- | --- | --- | --- | --- | --- | --- | --- | --- | --- | --- | --- | --- | --- | --- | --- | --- | --- | --- | --- | --- | --- | --- | --- | --- | --- | --- | --- | --- | --- | --- | --- | --- | --- | --- | --- | --- | --- | --- | --- | --- | --- | --- | --- | --- | --- | --- | --- | --- | --- | --- | --- | --- | --- | --- | --- | --- | --- | --- | --- | --- | --- | --- | --- | --- | --- | --- | --- | --- | --- | --- | --- | --- | --- | --- | --- | --- | --- | --- | --- | --- | --- | --- | --- | --- | --- | --- | --- | --- | --- | --- | --- | --- | --- | --- | --- | --- | --- | --- | --- | --- | --- | --- | --- | --- | --- | --- | --- | --- | --- | --- | --- | --- | --- | --- | --- | --- | --- | --- | --- | --- | --- | --- | --- | --- | --- | --- | --- | --- | --- | --- |
