# Supplementary material for: Mental well-being and work capacity: a cross-sectional study in a sample of the Swedish working population
Source: BMC Public Health. 2025 Sep 9;25:3046. doi: 10.1186/s12889-025-24015-1 (PMC12418673; doi:10.1186/s12889-025-24015-1)
Supplement: Supplementary file 6 — Supplementary Material 6. [file 12889_2025_24015_MOESM6_ESM.docx]

| **Additional file 6**. Univariable linear regression analysis of capacity to work and associated factors in a cohort based on the Swedish “Work Participation and Mental Health at Work” (ADAPT) research project, 2021 – 2022, stratified by gender (men: *n*=3557).   \| **Variables** \| **B** \| **95% CI** \| \| --- \| --- \| --- \| \| *WHO-5 Mental Well-being Index* (range, 0–100) \| –0.281 \| (–0.292;–0.270) \| \| *Age groups* \|  \|  \| \| 18 – 34 years (Ref.) \|  \|  \| \| 35 – 54 years \| –1.685 \| (–2.400;–0.970) \| \| 55 – 74 years \| –3.503 \| (–4.300;2.706) \| \| *Education level* \|  \|  \| \| University or higher (≥16 years) (Ref.) \|  \|  \| \| Post secondary (13–15 years) \| 0.754 \| (–0.205;1.713) \| \| Upper secondary (10–12 years) \| –0.305 \| (–0.962;0.353) \| \| Lower secondary or less (≤9 years) \| –0.783 \| (–1.664;0.098) \| \| *Occupational classification* \|  \|  \| \| Non-manual, high-skilled (Ref.) \|  \|  \| \| Non-manual, low-skilled \| –0.840 \| (–2.878;1.197) \| \| Manual, high-skilled \| –0.092 \| (–0.835;0.652) \| \| Manual, low skilled \| 2.405 \| (1.620;3.269) \| \| *Managerial position* \|  \|  \| \| Yes (Ref.) \|  \|  \| \| No \| –0.050 \| (–0.685;0.586) \| \| *Working time* \|  \|  \| \| Full-time (Ref.) \|  \|  \| \| Part–time \| 2.558 \| (1.533;3.584) \| \| *SF-36 General Health* \|  \|  \| \| Good/very good (Ref.) \|  \|  \| \| Moderate \| 6.947 \| (6.334;7.559) \| \| Poor/Very poor \| 13.843 \| (12.759;14.926) \| \| *Long–term mental health condition* \|  \|  \| \| No (Ref.) \|  \|  \| \| Yes \| 11.056 \| (10.168;11.945) \| \| *Work sector* \|  \|  \| \| Private (Ref.) \|  \|  \| \| Public \| 1.365 \| (0.782;1.948) \| \| Higher scores on the outcome indicate a more strained work capacity.  Abbreviations: B, Unstandardized Coefficient. CI, Confidence Interval. Ref., Reference category. \| \| \| \| |
| --- | --- | --- | --- | --- | --- | --- | --- | --- | --- | --- | --- | --- | --- | --- | --- | --- | --- | --- | --- | --- | --- | --- | --- | --- | --- | --- | --- | --- | --- | --- | --- | --- | --- | --- | --- | --- | --- | --- | --- | --- | --- | --- | --- | --- | --- | --- | --- | --- | --- | --- | --- | --- | --- | --- | --- | --- | --- | --- | --- | --- | --- | --- | --- | --- | --- | --- | --- | --- | --- | --- | --- | --- | --- | --- | --- | --- | --- | --- | --- | --- | --- | --- | --- | --- | --- | --- | --- | --- | --- | --- | --- | --- | --- | --- | --- | --- | --- | --- | --- | --- |
